# Metaphyseal bands in osteogenesis imperfecta

**DOI:** 10.4103/0971-3026.59752

**Published:** 2010-02

**Authors:** SS Suresh, John K Thomas

**Affiliations:** Department of Orthopaedics, Ibri Regional Referral Hospital, PO Box 46, Ibri 516, Al-Khod, Sultanate of Oman; 1 Department of Sultan Qaboos University Hospital, Al-Khod, Sultanate of Oman

**Keywords:** Osteogenesis imperfecta, pamidronate, zebra lines

## Abstract

An increasing number of patients with osteogenesis imperfecta are undergoing pamidronate therapy to prevent the incidence of fragility fractures. The authors herein report a child aged 3 years who received five cycles of pamidronate, resulting in metaphyseal bands, known as “zebra lines.”

## Introduction

Osteogenesis imperfecta, a disease characterized by brittle bones, is caused by a defect in the amount or structure of Type I collagen. The disease is characterized by fragility fractures, which may sometimes occur even during normal handling of the child by the mother, in severe forms of the disease. Although there is no specific treatment for the condition, various management strategies have evolved over the years in an attempt to improve the quality of bone and to improve the well being of the patient. These include the use of calcium supplementation, calcitonin and oral nitrogen-containing bisphosphonates.[1] As oral bisphosphonates cause gastric irritability, physicians have started using intravenous bisphosphonates. The beneficial effects of intravenous pamidronate in osteogenesis imperfecta were first reported by Astrom and Soderhall in 1993.[2–4]

## Case Report

**Figure 1:**
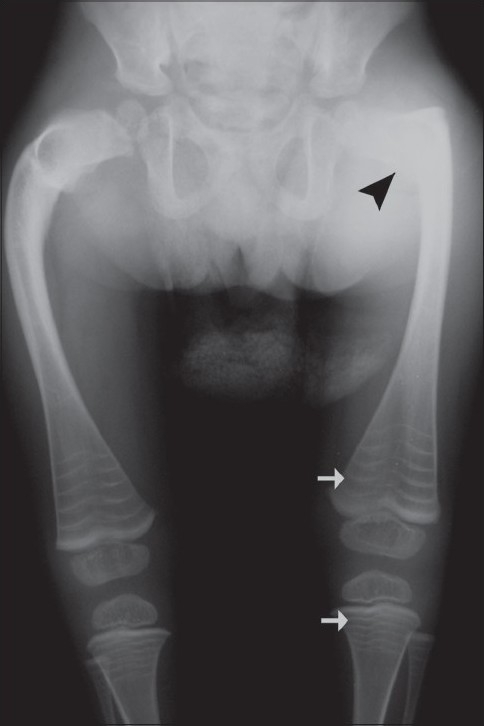
Frontal radiograph of both femurs shows a subtrochanteric fracture (black arrow head) with metaphyseal lines (zebra lines) involving both distal femoral and proximal tibial metaphyses (arrows)

Our case was a 3-year-old child, a known case of osteogenesis imperfecta type III, who presented with a history of multiple fragility fractures. Both lower extremities were deformed due to these fractures. In view of the osteopenia and the susceptibility to fractures, the child was started on intravenous pamidronate. The child received a total of five cycles of pamidronate 1.5 mg/kg body weight/day in three-day cycles. The cycles were repeated at an interval of 3 months. The child presented in the orthopedic department with a pathological fracture of the right subtrochanteric region due to trivial trauma [Figure 1].

**Figure 2:**
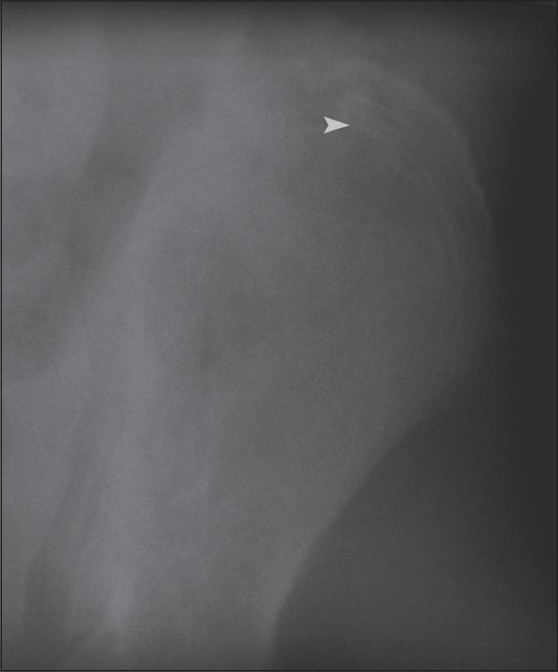
Frontal radiograph of the pelvic bones shows zebra lines involving the iliac crest (white arrow head)

**Figure 3:**
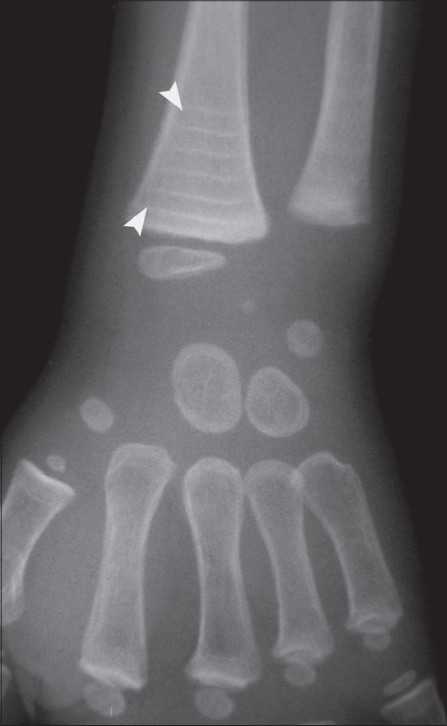
Posteroanterior radiograph of the wrist shows zebra lines (white arrow heads)

**Figure 4:**
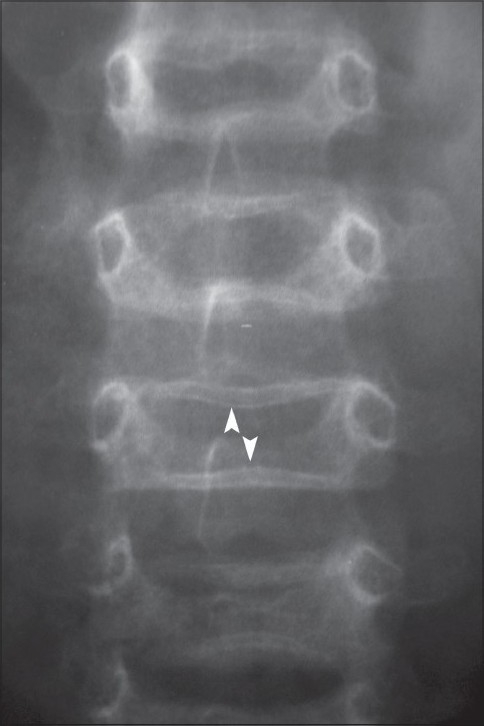
Frontal radiograph of the lumbar spine showing metaphyseal bands (white arrow heads)

Radiographs of the lower extremities revealed transverse sclerotic bands in the metaphyses of the proximal tibia and fibula as well as the distal femur [Figure 1]. Five bands were visible in the iliac metaphysis as well and there were transverse bands in the proximal femur and distal tibia [Figure 2]. Similar abnormalities were also seen in the wrist [Figure 3] and the spine [Figure 4].

## Discussion

Bisphophonates are widely used in the management of children with osteogenesis imperfecta to reduce the incidence of fractures and subsequent deformity. Because of the associated gastric intolerance, researchers started using intravenous pamidronate after Astrom and Soderhall[4] presented their first series of cases in 1993, showing successful management of osteogenesis imperfecta with pamidronate. Since then, there have been many reports of the usefulness of pamidronate therapy in moderate-to-severe osteogenesis imperfecta, resulting in a reduced rate of fractures and deformity.[2–4]

With the increasing use of bisphosphonates, there have been reports of abnormal radiological findings in the growing skeleton. Sclerosis of the epi-, apo- and metaphyseal areas of the appendicular and axial skeleton has been reported due to the administration of nitrogen-containing bisphosphonates,[1] with band-like areas of increased opacity in the growing bones. This is most marked in the distal metaphysis of the femur and proximal metaphyses of the tibia and fibula.[1,5]

Osteoclastic activity is inhibited during the cycle of pamidronate therapy, resulting in increased bone mineralization, which is seen on the radiographs as narrow lines parallel to the growth plate. The bands are the result of the failure of remodeling of the primary spongiosa into the secondary spongiosa in the physis. Further growth of the physis results in the appearance of normal bone, which results in the bands. Muderis *et al*. coined the term “zebra lines” for these radiographic findings.[5] The lines tend to be perpendicular to the axis of growth and span the width of the bone. In areas with slow growth, the lines are usually finer and more densely spaced.

The patterns of these zebra lines depend on the number of doses of intravenous pamidronate, the frequency of administration, the growth of the child and the bone studied. The number of lines correspond to the number of cycles of treatment the child has received,[3] with the lines being closer if the patient has received frequent doses. The distance between the zebra lines correlates with the rate of bone growth and the age of the child. However, the lines are seen only in children during the growing age, whereas the lines merge into one another in children nearing the prepubertal growth spurt. Zebra lines progressively move away from the physis, indicating growth disturbance in the physis, and disappear as they reach the diaphysis. The bands are seen as early as 2 months after the first treatment.[2] Metaphyseal bands are found to migrate away into the diaphysis on discontinuation of therapy. The bands stop appearing after physeal closure, even if therapy is continued.[1,5]

It has also been observed that pamidronate therapy does not prevent the future occurrence of fractures, although a sense of well being does increase in patients on pamidronate therapy,[2,4] along with a marked reduction in chronic bone pain.[3] Fractures continue to occur in osteogenesis imperfecta patients with improved mobility and greater activity,[2,3] because the bones are not structurally stronger. Indeed, the sense of well being due to treatment may make these patients more activethus resulting in an increased incidence of fractures.[2,4]

Faint metaphyseal lines seen in untreated children are called growth recovery lines. Transverse bands are also noted in heavy metal intoxication (lead), treated leukemia, healing rickets and chronic anemia; however, the sclerosis is not generalized and is more marked in the diaphysis.[1] In these situations, there is a period of growth suppression and subsequent recovery, which, if repetitive, results in multiple growth arrest lines.[6]
